# An ensemble-based approach for estimating personalized intraocular lens power

**DOI:** 10.1038/s41598-021-02288-x

**Published:** 2021-11-25

**Authors:** Salissou Moutari, Jonathan E. Moore

**Affiliations:** 1 School of Mathematics and Physics, Queens University Belfast University Road, Belfast, BT7 1NN Northern Ireland UK; 2 Cathedral Eye Clinic, 89-91 Academy Street, Belfast, BT1 2LS Northern Ireland UK

**Keywords:** Outcomes research, Translational research, Computational science, Statistics, Optical physics

## Abstract

The fundamental difference between modern formulae for intraocular lens (IOL) power calculation lies on the single ad hoc regression model they use to estimate the effective lens position (ELP). The ELP is very difficult to predict and its estimation is considered critical for an accurate prediction of the required IOL power of the lens to be implanted during cataract surgery. Hence, more advanced prediction techniques, which improve the prediction accuracy of the ELP, could play a decisive role in improving patient refractive outcomes. This study introduced a new approach for the calculation of personalized IOL power, which used an ensemble of regression models to devise a more accurate and robust prediction of the ELP. The concept of cross-validation was used to rigorously assess the performance of the devised formula against the most commonly used and published formulae. The results from this study show that overall, the proposed approach outperforms the most commonly used modern formulae (namely, Haigis, Holladay I, Hoffer Q and SRK/T) in terms of mean absolute prediction errors and prediction accuracy i.e., the percentage of eyes within ± 0.5D and ± 1 D ranges of prediction, for various ranges of axial lengths of the eyes. The new formula proposed in this study exhibited some promising features in terms of robustness. This enables the new formula to cope with variations in the axial length, the pre-operative anterior chamber depth and the keratometry readings of the corneal power; hence mitigating the impact of their measurement accuracy. Furthermore, the new formula performed well for both monofocal and multifocal lenses.

## Introduction

Cataract surgery has consistently advanced technologically over the past 20 years in relation to surgical instruments, intraocular lens (IOL) designs as well as biometry techniques. Despite these advances, refractive surgical surprises still remain one of the most paramount concerns for surgeons, post IOL implantation.

In addition to various pre-operative measurements and surgical precautions, accurate calculation of the IOL power is the key factor to mitigate refractive surprises in cataract surgery. Modern formulae used to estimate the power of the IOL to be implanted are derived from thin lens geometrical optics, combined with various patient specific pre-operative measurements. However, the concept of thin lens geometrical optics requires the knowledge of the eventual post-operative position of the IOL behind the cornea. This position, also referred to as the effective lens position (ELP), is defined as the axial depth from the cornea to the optical center of the IOL. Since, the ELP is not measurable pre-operatively, modern formulae for IOL power calculation resort to various prediction techniques to estimate it. An accurate prediction of the ELP is considered to be crucial for the calculation of the required power for a given IOL. However, the ELP is prone to significant variations between different eyes. These variations may be attributed to various factors, including patient anatomical and physiological factors, IOL design as well as surgical techniques.

The most widely used formulae to estimate the IOL power, and which are currently the industry norms and are published in an implementable form, include the Haigis formula^1^, the Hoffer Q formula^2^, the Holladay I formula^3^ and the SRK/T formula^4^. The last three formulae used two pre-operative measurements, namely the axial length of the eye and the average corneal power,—derived from the average keratometry readings in diopters, which are combined with an additional ad hoc constant (pACD—“personalized” Anterior Chamber Depth—for Hoffer Q, SF—Surgeon Factor—for Holladay I, and A_constant_ for SRK/T) associated with each IOL type, and also referred to as the IOL constant, to estimate the required power for a given IOL. On the other hand, the Haigis formula^1^ used three pre-operative measurements (namely, the axial length of the eye, the average corneal power and the pre-operative measured anterior chamber depth), which are used to estimate the three constants of the formula (a_0_, a_1_, a_3_) associated with each IOL type.

Other well-known formulae for IOL power calculation include the Olsen formula^5^, the Barrett Universal II formula^6^ and the Holladay II formula; however, the last two formulae have not been published and the details regarding their implementation are not available. Recently, IOL power calculation formulae based on machine learning predictive models have been introduced. The models used in the implementation of these formulae include neural networks^7–9^ and Bayesian additive regression tree^10^. However, the details on the implementation process of these formulae are not available.

The fundamental difference between modern formulae for IOL power calculation lies in the prediction model used to estimate the ELP. Basically, each formula used a single ad hoc regression model, generally devised from clinical experience and manipulation of retrospective empirical data, to predict the ELP. Hence, some formulae tend to outperform others depending on the type of pre-operative measurements and/or the cohort of patients, leading to the high disparity in the conclusions drawn in many analyses comparing formulae for IOL power calculation, e.g.,^11–16^. In view of the high sensitivity of the ELP, stemming from the complex relationship with its potential influencing factors, advanced prediction techniques would be the most natural approach for improving the prediction accuracy of the ELP.

This study proposed a new approach for IOL power estimation (called the MM formula), which used an ensemble of regression models to obtain a more accurate prediction of the ELP. Hence, as opposed to most of the modern IOL power calculation formulae, the MM formula has a very high number of IOL constants, which are optimally devised, stored and managed through the machine learning ensemble model used.

## Methods

### Background

The most commonly known IOL power calculations formulae can be categorized into two main approaches: the first one is purely based on a linear regression analysis of retrospective cases, whereas the second one is based on a geometrical optics solution. The first IOL power calculation formulae^17–19^, based on linear regression, are purely statistical solution and not in use in clinical practice today. These formulae suffer from classical linear regression shortcomings, including the regression to mean problem. In other words, the more common the eye’s characteristics, the more accurate the predicted power, while unusual eyes result in very poor estimates of the power.

The first IOL power calculation formulae, based on geometrical optics^20–24^, consist of different variants of the following vergence formula (1), derived from a two-lens system (eye-IOL) model of an operated eye after cataract removal and insertion of an IOL.

1 $${\text{P}} = {{\text{n}}_{\text{vit}}} ~/\left( {{\text{AL}} - {\text{d}}} \right)~ - {{\text{n}}_{{{\text{aq}}}}} /\left[ {\left( {{{\text{n}}_{{{\text{aq}}}}} /{{\text{P}}_{{\text{c}}}} } \right) - {\text{d}}} \right],$$

where P is the required IOL power for emmetropia (in diopters), n_aq_ is the refractive index of the aqueous humor and n_vit_ is the refractive index of the vitreous humor, P_c_ is the average corneal power (in diopters) and is a function of the average keratometry readings K = (K_1_ + K_2_)/2, AL is the axial depth from the corneal apex to the retina, also known as the axial length of the eye, d is the axial depth from the corneal apex to the optical center of the IOL, also known as effective/estimated lens position (ELP) or the post-operative Anterior Chamber Depth (ACD).

Initially, all the variants of the formula (1) used a constant value for the ELP, d, for each IOL type, which is derived using the parameters (K and AL) of an average eye. This constant value of the ELP is also known as the ACD constant. In the early eighties new studies observed some inter-subject variation in the value of the ELP, and in particular the available formulae proved to be deficient for eyes with unusually short or long axial lengths. The variation in the ELP can be attributed to various factors, including:

- Patient-specific anatomical and physiological factors such as ocular dimensions, age, gender, and ethnicity;
- IOL design-specific configuration details such as the optical shape factor, the compressibility of materials, and the haptic angulation;
- Surgeon as well as surgical instrument and technique specific idiosyncrasies such as the IOL implantation location (e.g., angle-supported, iris-supported, sulcus- supported, or in-the-bag), the manipulation of the IOL during implantation, the type, the size, and the structure of the incision, as well as the size, the construction (manual or automated), and the configuration of the capsulorhexis.

In the late eighties, new formulae using a patient-specific modified ELP value, which considered biometry values specific to an individual patient for a particular IOL, emerged. These new formulae are referred to as modern IOL formulae^1–6^.

Modern formulae for IOL power estimation, such as Haigis^1^, Hoffer Q^2^, Holladay I^3^ and SRK/T^4^ are based on the vergence Formula (2) derived from a three-lens system (spectacle-eye-IOL), and they differ from each other merely in the approach used to estimate the effective lens position, d.

2 $${\text{P}} = {{\text{n}}_{{{\text{vit}}}}} ~/\left( {{\text{AL}} - {\text{d}}} \right)~ - {{\text{n}}_{{{\text{aq}}}}} /\left[ {\left( {{{\text{n}}_{{{\text{aq}}}}} /\left( {{{\text{ P}}_{{\text{c}}}} ~ + {{\text{R}}_{{\text{x}}}} /\left( {{\text{1}} - {\text{b}}\, {{\text{R}}_{{\text{x}}}} } \right)} \right)} \right) - {\text{d}}} \right],$$

where R_x_ is the desired postoperative refraction (in diopters) or the refraction of the spectacle, P is the required IOL power for the desired postoperative refraction (in diopters), b is the vertex distance (~ 12 mm), n_aq_, n_vit_, P_c_, L and d, are as defined in Eq. (1).

The ELP is very difficult to predict, and its estimation is considered critical for an accurate prediction of the required IOL power for a given lens. The main idea behind modern formulae, such as^1–6^, is to improve the IOL power accuracy by estimating the ELP, d, through a regression analysis on retrospective cases. For this methodology to be effective, consistency is required throughout the entire process, including the surgical technique used, the biometry instruments as well as the design and manufacturing of the IOL.

The main adjustment to the geometrical optics Formula (2), by the modern formulae, was the estimation of the ELP as a function of pre-operative measurements, such as the axial length in millimeters (AL), corneal power in diopters, derived from the keratometry readings (K_1_ and K_2_), and the anterior chamber depth (ACD).

The most commonly used and well-known modern formulae for IOL power calculation include Haigis^1^, Hoffer Q^2^, Holladay I^3^ and SRK/T^4^. These formulae were published during the 1990s, and are currently the industry norm. The Hoffer Q formula estimates the ELP as a sum of an ad hoc constant, called the "personalized" ACD and denoted pACD, and a function of the average kerotometric readings and the axial length of the eye. The Holladay I formula estimates the ELP as a sum of an ad hoc constant, called the surgeon factor and denoted SF, and a function of kerotometric readings and the axial length of the eye. The SRK/T formula estimates the ELP as the sum of a scaled constant, denoted A_constant_, and a function of the average kerotometric reading and the axial length of the eye. The Haigis formula went one step further, by including the pre-operative ACD in the estimation of the ELP, and uses three regression constants denoted (a_0_, a_1_, a_2_).

The constants used in these formulae, namely, pACD, SF, A_constant,_ and (a_0_, a_1_, a_2_) are derived from historical data of retrospective cases and are expected to capture the complex relationship for each IOL/surgeon pair.

### A new formula using an ensemble-based model to estimate the ELP

Although the main aim of modern formulae is to “personalize” the IOL power calculation through linear regression models for predicting the ELP where the parameters of the models are a_0_, a_1_, a_2_ for Haigis, pACD for Hoffer Q, SF for Holladay, and A_constant_ for SRK/T), in practice the parameters of the models (also referred to as the lens constants) are initially made available by the lens manufacturer and subsequently optimized and published on some databases such as ULIB^25^, using data from various surgeons or a selected number of surgeons. On the other hand, it is well recognized that current modern formulae still demonstrate significant errors in the prediction of IOL power for unusual cases with extreme values of either axial length or corneal power^26^. For example, in short eyes with flat cornea, and long eyes with a steep cornea, the discrepancy can be up to ± 2D and ± 1.3D, respectively.

This newly developed MM formula, which leverages both thin lens geometric optics and machine learning, goes a step further in an attempt to reduce the effects of these discrepancies, by introducing an ensemble-based approach to estimate the effective lens position, for each lens model using four pre-operative variables, namely the steep and the flat keratometry readings, the pre-operative anterior chamber depth, and axial length of the eyes. These variables are used as predictor to estimate the ELP using a high-dimensional function, derived by training a machine learning ensemble-based model. The four predictor variables, used to estimate the ELP, have been identified through a feature selection approach, and are deemed to be the most influential variables in the prediction of the ELP, hence they are expected to capture both surgeon as well as surgical instrument and technique specific idiosyncrasies. In contrast with the single linear regression model, which is commonly used to estimate the ELP^1–4^, machine learning is the most natural tool able to capture the complex relationship between the ELP, the post-operative patient data, the surgeon as well as features specific to surgical instruments and techniques for each lens model. Furthermore, the proposed model to predict the ELP and calculate the IOL power, is not only surgeon-specific but is also self-sustained since the more the historical data available the more “personalized” and accurate the IOL power estimation.

**Figure 1:**
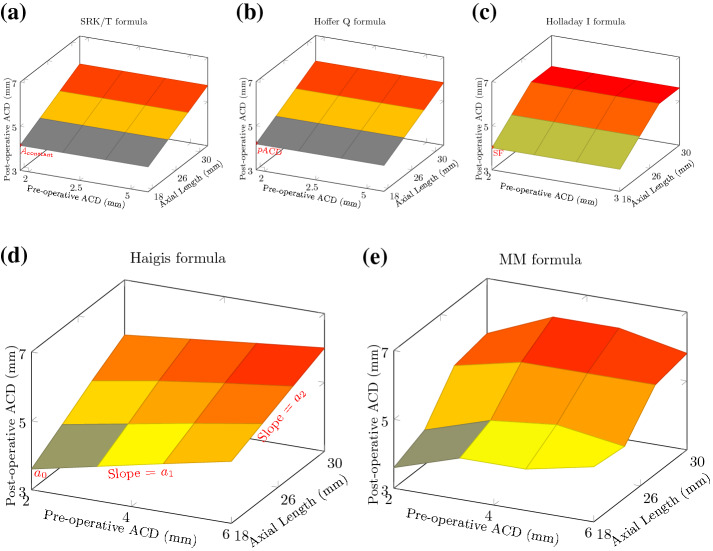
A comparative illustration of the relationship between the post-operative ACD (i.e., ELP) and the measured pre-operative ACD and the axial length using the formulae SRK/T, Hoffer Q, Holladay I, Haigis and MM, for a given lens and some given keratometry readings K_1_ and K_2_.

The major difference between the MM formula and the four most commonly used formulae can be summarized as follows. For a given lens model and some given keratometry readings (K_1_ and K_2_), the Hoffer Q and SRK/T formulae consider a quasi-linear relationship between the post-operative ACD (i.e., ELP) and the axial length, whereas the Holladay I formula assumes a piecewise linear relationship between the post-operative ACD (ELP) and the axial length, regardless of any other measurements including the pre-operative ACD, as illustrated in Fig. 1 (top). On the other hand, the Haigis formula assumes that the post-operative ACD (ELP) depends linearly on the pre-operative measured ACD and the axial length while the MM formula assumes a non-linear relationship between the post-operative ACD (ELP) and the following variables: the pre-operative ACD and the axial length, as illustrated in Fig. 1 (bottom). Therefore, unlike most of the modern formulae for IOL power calculation, which used one or three IOL constants, the MM formula has a very high number of IOL constants. However, these constants are optimized, stored and managed automatically via the ensemble model used. The MM formula differs from the other machine learning models for IOL power calculation^7–10^, by combining both geometric optics and machine learning to estimate the ELP.

### Assessment of the proposed formula

To carry out a rigorous comparison between the MM formula and the four most commonly used formulae, namely Haigis, Hoffer Q, Holladay I, and SRK/T, we used the same data set to train the ensemble model for the MM formula and optimize the IOL constants for the four formulae, i.e., the three regression parameters (a_0_, a_1_, a_2_) for the Haigis formula, the personalized anterior chamber depth—pACD—for the Hoffer Q formula, the surgeon factor—SF—for the Holladay I formula, and the A_constant_ for the SRK/T formula.

Most of the studies, comparing formulae for IOL power calculation, used a holdout method, which consists of splitting the data into two sets, namely the training set and the test set, respectively. The training data set referred to patients’ data used to optimize the parameters of the formulae whereas the test set, referred to patients’ data not included in the optimization process. The prediction errors made using the test set are used to evaluate the performance of the formula. However, such an evaluation process may have a high variance, since it depends heavily on the nature of the data in both the training and the test sets. Therefore, this approach of comparing formulae for IOL power calculation, is prone to bias since it may differ significantly depending on the data, which happened to be in the test set.

One way to address the aforementioned limitations of the handout method is to use the cross-validation technique, also known as the k-fold cross-validation. The cross-validation technique is the most effective framework to assess how a predictive model generalizes to independent datasets. It enables to generate both training and test samples, which are sufficiently large and diverse in order to be representative. As such, it addresses not only the problem of the small sample size of eyes with short and long axial length, in most of the study cohorts, but also enable to assess the formulae on a variety of training and test sets. Hence, it is the most appropriate approach to assess the performance of IOL calculation formulae, which are essentially predictive models.

**Figure 2:**
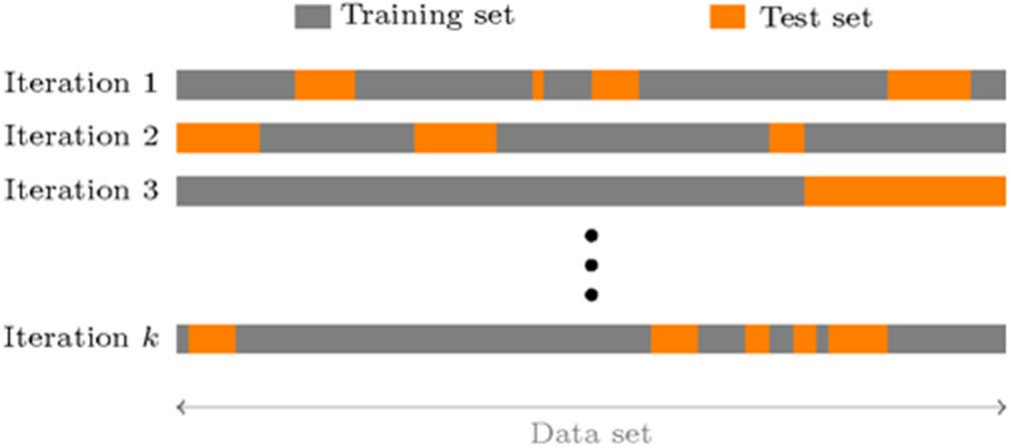
Illustration of the concept of k-fold cross-validation.

In the k-fold cross-validation approach, the data set is split into k subsets, and the holdout method is applied k times as follows: at each step, (k − 1) subsets are combined to form the training set whereas the remaining dataset is used as the test set. Then, the prediction errors made during the test are given by the accumulated errors from the k trials. Another variant of the k-fold cross-validation method consists of randomly splitting the data into training and test sets (k-fold) and the handout method is applied at each iteration. Such an approach, also known as the Monte-Carlo cross-validation, and illustrated in Fig. 2, has been used in this study as it enables to assess how well an IOL power calculation formula will generalize to new data.

### Participants

The participants consist of a cohort of 681 patients who had implantation of monofocal or multifocal IOLs from Cathedral Eye Clinic, Belfast. More specifically, 265 eyes, 256 eyes and 160 eyes were implanted with Monofocal Alcon AcrySofIQ SN60WF, Monofocal Lenstec Softec HDO and Multifocal Zeiss AT LISA tri839 MP, respectively. Patients were thoroughly assessed and informed of the risks of the procedure and all patients gave their informed consent for their anonymized data to be used for audit and research purposes. The Cathedral Eye Clinic Ethics Committee approved this study as an audit study and gave the study the following reference number: CECREC18–02. Furthermore, research study adhered to the tenets of the Declaration of Helsinki. The patients received multifocal IOLs following either refractive lens exchange (RLE) or cataract extraction surgery. The summary statistics of the patients are presented in Table 1 and in the supplementary material (Table S1). The post-operative data used for this study included manifest refraction obtained 3 months and 6 months post-operatively, and included only one eye per patient.

**Table 1:** Summary of demographics and biometry.

Demographics and biometry							
Lens model	Sample Size (Eyes) One eye per patient	Gender% Female %Male	Age (in years) Mean ± SD MedianRange	AL (in mm) Mean ± SD Median Range	K_1_ (in D) Mean ± SD Median Range	K_2_ (in D) Mean ± SD Median Range	ACD (in mm) Mean ± SD MedianRange
Monofocal Alcon AcrySofIQ SN60WF	265	52% 48%	66 ± 10 67 36–90	23.80 ± 1.53 23.49 20.50–29.63	43.06 ± 1.83 43.19 35.56–47.20	43.84 ± 1.94 43.83 36.06–48.70	3.24 ± 0.41 3.00 2.17–4.80
Monofocal Lenstec Softec HDO	256	50% 50%	71 ± 10 73 45–94	23.59 ± 1.42 23.46 20.06–29.37	43.25 ± 1.65 43.38 35.70–46.88	44.03 ± 1.69 44.12 36.20–48.49	3.21 ± 0.39 3.00 2.33–4.22
Multifocal ZEISS AT LISA tri839 MP	160	60% 40%	58 ± 7 58 44–92	23.55 ± 1.32 23.42 20.48–27.85	42.86 ± 1.50 42.72 38.38–47.34	43.50 ± 1.54 43.47 38.96–47.77	3.22 ± 0.35 3.00 2.37– 4.22

*AL* Axial Length, *K*_*1*_ Flat Keratometry readings, *K*_*2*_ Steep Keratometry readings, *SD* Standard Deviation.

### Statistical analysis

The prediction error (PE), for a given patient, is the difference between the spherical equivalent of the achieved post-operative refraction and the pre-operative predicted refraction obtained using a formula, given the power of the implanted IOL.

Throughout the analysis, we have also used the following abbreviations: SD is standard deviation of the prediction error, MedAE is the median absolute prediction error, MAE is the mean absolute prediction error.

The normality of the prediction errors as well as the absolute prediction errors for each of the formula and for each eye type are assessed using the Shapiro–Wilk normality test, which suggested that none of them is normally distributed (*p*-value < 0.001 in all the cases). Therefore, non-parametric tests, with the significance level set to 0.05, are used for the statistical analysis. Wilcoxon signed-rank (1 sample) test was used to assess whether the median values for both the prediction errors and the absolute prediction errors are equal to zero for each of the formula and for each eye type. The test results for the median of the prediction errors are presented in the supplementary material (Tables S3a, S4a, S5a). The test results for the median of the absolute prediction errors suggested that in all cases the median value is statistically different from zero (*p*-value < 0.001 in all case).

The Friedman test was used to compare the median absolute prediction error across the five formulae for each eye type. The results of test suggested that in all cases there was a statistically significant difference across the formulae. Then, the pairwise comparison of the median absolute prediction error (MM against each of the other four formulae, and for each eye type) was performed using the Wilcoxon signed-rank (paired samples). The corresponding test results as well as those of the Friedman test are presented in the supplementary material (Tables S3b, S4b, S5b).

The Cochran’s Q test was used to compare the prediction accuracy across the five formulae for each eye type. The prediction accuracy is defined by the percentage of eyes with the prediction error within the range ± 0.5D, ± 1.0D, and ± 1.5D, respectively. The corresponding test results are presented in the supplementary material (Tables S3, S4c, S5c). The pairwise comparison of the prediction accuracy (MM against each of the four other formulae, and for each eye type) was performed using the McNemar test. The corresponding test results are presented in Tables 4, 7, 10.

The implementation of the formulae as well the statistical analyses were carried out using Python 3.7.6 (python.org).

## Results

To assess the performance of the MM formula, three lens models were considered, namely Monofocal Alcon AcrySofIQ SN60WF, Monofocal Lenstec Softec HDO and Multifocal Zeiss AT LISA tri839 MP. Summary statistics of the optimized IOL constants, for the four formulae and for each of the three lens models, obtained using the Monte-Carlo k-fold cross-validation process (with k = 100), are presented in the supplementary material (Table S2).

The performance of the MM against the other four formulae, with respect to the axial length, was assessed, using the following categorization of the eyes: short eyes (i.e., Axial Length < 22 mm), medium eyes (i.e., 22 mm <  = Axial Length <  = 24.5 mm), long medium eyes (i.e., 24.5 mm < Axial Length <  = 26 mm) and long eyes (i.e., Axial Length > 26 mm).

Tables 2, 3, 5, 6, 8, 9 present the summary statistics of the cross-validation prediction results for each of the five formulae (SRK/T, Hoffer Q, Holladay I, Haigis and MM) for long eyes, long medium eyes, short eyes, medium eyes and all eyes, respectively. These results were obtained using the optimized IOL constants for each of the formulae, presented in the supplementary material (Table S2), and the same data used to optimize these IOL constants were used to train the MM formula and store the corresponding ensemble model.

**Table 2:** Summary statistics of the cross-validation results for each of the five formulae (SRK/T, Hoffer Q, Holladay I, Haigis and MM) for long, long medium and short eyes.

Monofocal Alcon AcrySofIQ SN60WF Summary statistics of cross − validation results for long, long medium and short eyes					
**Long eyes** (Bootstrap sample size: 282)					
Statistics	SRK/T	Hoffer Q	Holladay I	Haigis	MM
% Rx within ± 0.5D	23.40%	40.80%	39.00%	**62.40%**^a^	**64.20%**^a^
% Rx within ± 1.0D	51.80%	80.90%	72.70%	**92.20%**^a^	**92.20%**^a^
% Rx within ± 1.5D	79.10%	92.20%	88.30%	**100.00%**^a^	**100.00%**^a^
MPE ± SD	− 0.89 ± 0.86	0.59 ± 0.55	0.74 ± 0.63	0.26 ± 0.52	0.16 ± 0.51
MedPE	− 0.92	0.6	0.7	0.19	0.03
Range PE	− 2.51 to 1.13	− 0.28 to 1.96	− 0.27 to 2.35	− 0.63 to 1.49	− 0.65 to 1.33
MAPE ± SD	1.05 ± 0.67	0.66 ± 0.47	0.78 ± 0.58	0.47 ± 0.36	0.41 ± 0.34
MedAPE	0.97	0.6	0.7	0.4	0.35
**Long medium eyes** (Bootstrap sample size: 452)					
Statistics	SRK/T	Hoffer Q	Holladay I	Haigis	MM
% Rx within ± 0.5D	19.90%	80.10%^a^	**87.60%**	77.70%	82.10%^a^
% Rx within ± 1.0D	32.30%	95.40%	**100.00%**^a^	**100.00%**^a^	**100.00%**^a^
% Rx within ± 1.5D	50.20%	**100.00%**^a^	**100.00%**^a^	**100.00%**^a^	**100.00%**^a^
MPE ± SD	− 1.31 ± 0.99	0.22 ± 0.38	− 0.04 ± 0.35	− 0.01 ± 0.39	− 0.09 ± 0.35
MedPE	− 1.49	0.26	− 0.14	**0**^b^	− 0.07
Range PE	− 2.99 to 0.83	− 0.61 to 1.04	− 0.7 to 0.79	− 0.8 to 0.94	− 0.87 to 0.79
MAPE ± SD	1.41 ± 0.85	0.36 ± 0.25	0.3 ± 0.19	0.3 ± 0.25	0.28 ± 0.23
MedAPE	1.49	0.38	0.27	0.25	**0.21**
**Short eyes** (Bootstrap sample size:218)					
Statistics	SRK/T	Hoffer Q	Holladay I	Haigis	MM
% Rx within ± 0.5D	65.60%	59.60%^a^	**66.10%**	60.60%	56.90%^a^
% Rx within ± 1.0D	89.00%	**100.00%**	95.40%^a^	89.00%	93.60%^a^
% Rx within ± 1.5D	**97.70%**^a^	**100.00%**^a^	**100.00%**^a^	**100.00%**^a^	**97.70%**^a^
MPE ± SD	0.08 ± 0.6	− 0.22 ± 0.46	− 0.22 ± 0.49	0.08 ± 0.55	0.17 ± 0.58
MedPE	0.15	− 0.3	− 0.27	− **0.03**^b^	0.12
Range PE	− 1.18 to 1.59	− 0.78 to 0.94	− 0.99 to 1.04	− 0.86 to 1.42	− 0.91 to 1.54
MAPE ± SD	0.44 ± 0.41	0.45 ± 0.24	0.46 ± 0.28	0.42 ± 0.36	0.45 ± 0.4
MedAPE	**0.28**^a^	0.4	0.39	0.42	**0.28**^a^

*% Rx* Percentage of eyes within a given range of prediction error, *MPE* Mean prediction error, *MedPE* Median prediction error, *Range PE* Range of the prediction error, *MAPE* Mean absolute prediction error, *MedAPE* Median absolute prediction error, *SD* Standard Deviation of the error.

^a^No statistically significant difference at level 0.05.

^b^No statistically significant difference from zero at level 0.05.

Significant values are in [bold].

**Table 3:** Summary statistics of the cross-validation results for each of the five formulae (SRK/T, Hoffer Q, Holladay I, Haigis and MM) for medium and all eyes.

Monofocal Alcon AcrySofIQ SN60WF Summary statistics of cross-validation results for medium and all eyes					
**Medium eyes** (Bootstrap sample size: 1534)					
Statistics	SRK/T	Hoffer Q	Holladay I	Haigis	MM
% Rx within ± 0.5D	68.80%	**74.30%**^a^	72.40%	72.60%	**75.70%**^a^
% Rx within ± 1.0D	94.10%	93.70%	92.90%	**96.10%**^a^	**96.40%**^a^
% Rx within ± 1.5D	**97.20%**^a^	**97.80%**^a^	**98.00%**^a^	**98.00%**^a^	**97.80%**^a^
MPE ± SD	0.05 ± 0.55	− 0.06 ± 0.54	− 0.17 ± 0.52	− 0.05 ± 0.52	− 0.03 ± 0.52
MedPE	0.04	− 0.03	− 0.15	− 0.03	− 0.01^b^
Range PE	− 2.1 to 1.89	− 2.35 to 1.84	− 2.23 to 1.73	− 2.51 to 1.65	− 2.3 to 1.67
MAPE ± SD	0.42 ± 0.37	0.39 ± 0.38	0.4 ± 0.37	0.38 ± 0.36	0.37 ± 0.36
MedAPE	0.35	0.28	0.29	0.28	0.27
**All eyes** (Bootstrap sample size: 2486)					
Statistics	SRK/T	Hoffer Q	Holladay I	Haigis	MM
% Rx within ± 0.5D	54.50%	70.20%	70.80%	71.30%	**73.90%**
% Rx within ± 1.0D	77.60%	93.10%	92.10%	95.70%	**96.30%**
% Rx within ± 1.5D	86.60%	97.80%	97.50%	**98.80%**	98.40%
MPE ± SD	− 0.3 ± 0.89	0.05 ± 0.56	− 0.05 ± 0.58	0.01 ± 0.52	− 0.0 ± 0.5
MedPE	− 0.06	0.05	− 0.12	**0**^b^	**0**^b^
Range PE	− 2.99 to 1.89	− 2.35 to 1.96	− 2.23 to 2.35	− 2.51 to 1.65	− 2.3 to 1.67
MAPE ± SD	0.67 ± 0.66	0.42 ± 0.37	0.43 ± 0.39	0.38 ± 0.35	0.36 ± 0.35
MedAPE	0.45	0.33	0.31	0.29	**0.27**

*% Rx* Percentage of eyes within a given range of prediction error, *MPE* Mean prediction error, *MedPE* Median prediction error, *Range PE* Range of the prediction error, *MAPE* Mean absolute prediction error, *MedAPE* Median absolute prediction error, *SD* Standard Deviation of the error.

^a^No statistically significant difference at level 0.05.

^b^No statistically significant difference from zero at level 0.05.

Significant values are in [bold].

**Table 4:** Pairwise comparison of the prediction accuracy (i.e., the percentage of eyes within a given range of prediction error) between the MM formula and each of the other four formulae (SRK/T, Hoffer Q, Holladay I and Haigis) for the different types of eyes (long, long medium, medium, short and all eyes), using the McNemar test at a statistical significance level of 5%.

Monofocal Alcon AcrySofIQ SN60WF			
Pairwise comparison of the prediction accuracy *p*-values for McNemar test			
**Long eyes**			
	% Rx within ± 0.5D	% Rx within ± 1.0D	% Rx within ± 1.5D
MM vs SRK/T	< **0.001**^b^	< **0.001**^b^	< **0.001**^b^
MM vs Hoffer Q	< **0.001**^b^	< **0.001**^b^	< **0.001**^b^
MM vs Holladay I	< **0.001**^b^	< **0.001**^b^	< **0.001**^b^
MM vs Haigis	0.603^a^	0.999^a^	0.999^a^
**Long medium eyes**			
	% Rx within ± 0.5D	% Rx within ± 1.0D	% Rx within ± 1.5D
MM vs SRK/T	< **0.001**^b^	< **0.001**^b^	< **0.001**^b^
MM vs Hoffer Q	0.435^a^	< **0.001**^b^	0.999^a^
MM vs Holladay I	*0.001*^c^	0.999^a^	0.999^a^
MM vs Haigis	< **0.001**^b^	0.999^a^	0.999^a^
**Medium eyes**			
	% Rx within ± 0.5D	% Rx within ± 1.0D	% Rx within ± 1.5D
MM vs SRK/T	< **0.001**^b^	< **0.001**^b^	0.523^a^
MM vs Hoffer Q	0.077^a^	< **0.001**^b^	0.999^a^
MM vs Holladay I	< **0.001**^b^	< **0.001**^b^	0.134^a^
MM vs Haigis	< **0.001**^b^	0.441^a^	0.134^a^
**Short eyes**			
	% Rx within ± 0.5D	% Rx within ± 1.0D	% Rx within ± 1.5D
MM vs SRK/T	< *0.001* ^c^	**0.004**^b^	0.752^a^
MM vs Hoffer Q	0.581^a^	*0.001* ^c^	0.074^a^
MM vs Holladay I	*0.019* ^c^	0.134^a^	0.074^a^
MM vs Haigis	*0.013* ^c^	**0.004**^b^	0.074^a^
**All eyes**			
	% Rx within ± 0.5D	% Rx within ± 1.0D	% Rx within ± 1.5D
MM vs SRK/T	< **0.001**^b^	< **0.001**^b^	< **0.001**^b^
MM vs Hoffer Q	< **0.001**^b^	< **0.001**^b^	**0.01**^b^
MM vs Holladay I	< **0.001**^b^	< **0.001**^b^	< **0.001**^b^
MM vs Haigis	< **0.001**^b^	**0.021**^b^	*0.008* ^c^

^a^No statistically significant difference at level 0.05.

^b^MM formula outperformed at significance level 0.05.

^c^MM formula underperformed at significance level 0.05.

Significant values are in [bold, Italics].

**Table 5:** Summary statistics of the cross-validation results for each of the five formulae (SRK/T, Hoffer Q, Holladay I, Haigis and MM) for long, long medium and short eyes.

Monofocal Lenstec Softec HDO Summary statistics of cross-validation results for long, long medium and short eyes					
**Long eyes** (Bootstrap sample size: 107)					
Statistics	SRK/T	Hoffer Q	Holladay I	Haigis	MM
% Rx within ± 0.5D	38.30%	37.40%	41.10%	67.30%	**72.90%**
% Rx within ± 1.0D	74.80%	**100.00%**^a^	**100.00%**^a^	**100.00%**^a^	**100.00%**^a^
% Rx within ± 1.5D	81.30%	**100.00%**^a^	**100.00%**^a^	**100.00%**^a^	**100.00%**^a^
MPE ± SD	− 0.79 ± 0.69	0.36 ± 0.53	0.46 ± 0.44	0.05 ± 0.49	0.01 ± 0.44
MedPE	− 0.52	0.41	0.64	0.21	**0.17**^b^
Range PE	− 2.34 to 0.01	− 0.74 to 0.99	− 0.51 to 0.94	− 0.97 to 0.66	− 0.96 to 0.56
MAPE ± SD	0.79 ± 0.69	0.6 ± 0.23	0.61 ± 0.2	0.41 ± 0.28	0.36 ± 0.25
MedAPE	0.52	0.69	0.64	0.36	**0.27**
**Long medium eyes** (Bootstrap sample size:306)					
Statistics	SRK/T	Hoffer Q	Holladay I	Haigis	MM
% Rx within ± 0.5D	24.50%	69.60%	**83.70%**^a^	**82.00%**^a^	**81.40%**^a^
% Rx within ± 1.0D	43.50%	**97.40%**^a^	**98.00%**^a^	**98.70%**^a^	**98.00%**^a^
% Rx within ± 1.5D	63.10%	**100.00%**^a^	**100.00%**^a^	**100.00%**^a^	**100.00%**^a^
MPE ± SD	− 1.08 ± 0.84	0.23 ± 0.41	0.07 ± 0.43	0.05 ± 0.39	0.07 ± 0.41
MedPE	− 1.33	0.23	− **0.04**^b^	0.01	**0**^b^
Range PE	− 2.43 to 0.73	− 0.78 to 1.11	− 0.63 to 1.41	− 1.03 to 1.01	− 0.91 to 1.3
MAPE ± SD	1.18 ± 0.7	0.38 ± 0.29	0.34 ± 0.27	0.33 ± 0.23	0.32 ± 0.27
MedAPE	1.33	0.28	0.31	0.3	0.25
**Short eyes** (Bootstrap sample size: 228)					
Statistics	SRK/T	Hoffer Q	Holladay I	Haigis	MM
% Rx within ± 0.5D	41.20%	**55.70%**^a^	43.40%	**53.10%**^a^	**53.10%**^a^
% Rx within ± 1.0D	64.00%	85.50%^a^	86.00%^a^	**93.90%**	84.20%^a^
% Rx within ± 1.5D	88.60%	**100.00%**	**100.00%**	**100.00%**	97.40%
MPE ± SD	0.54 ± 0.8	− 0.28 ± 0.55	− 0.09 ± 0.62	0.09 ± 0.62	0.08 ± 0.69
MedPE	0.52	− 0.29	− **0.02**^b^	**0.07**^b^	− **0.06**^b^
Range PE	− 0.74 to 2.14	− 1.23 to 0.79	− 1.13 to 1.13	− 1.0 to 1.42	− 1.11 to 1.7
MAPE ± SD	0.77 ± 0.58	0.52 ± 0.34	0.52 ± 0.36	0.52 ± 0.35	0.59 ± 0.36
MedAPE	0.63	**0.42**	0.54	0.43^a^	0.44^a^

*% Rx* Percentage of eyes within a given range of prediction error, *MPE* Mean prediction error, *MedPE* Median prediction error, *Range PE* Range of the prediction error, *MAPE* Mean absolute prediction error, *MedAPE* Median absolute prediction error, *SD* Standard Deviation of the error.

^a^No statistically significant difference at level 0.05.

^b^No statistically significant difference from zero at level 0.05.

Significant values are in [bold].

**Table 6:** Summary statistics of the cross-validation results for each of the five formulae (SRK/T, Hoffer Q, Holladay I, Haigis and MM) for medium and all eyes.

Monofocal Lenstec Softec HDO Summary statistics of cross-validation results for medium and all eyes					
**Medium eyes** (Bootstrap sample size: 1731)					
Statistics	SRK/T	Hoffer Q	Holladay I	Haigis	MM
% Rx within ± 0.5D	69.80%	71.20%	74.60%	70.40%	**78.70%**
% Rx within ± 1.0D	93.60%	95.50%	95.60%	96.00%	**96.90%**
% Rx within ± 1.5D	**99.70%**^a^	**99.70%**^a^	**99.50%**^a^	**99.70%**^a^	**99.70%**^a^
MPE ± SD	0.24 ± 0.46	− 0.02 ± 0.49	− 0.07 ± 0.45	− 0.02 ± 0.48	− 0.02 ± 0.45
MedPE	0.23	− **0.02**^b^	− 0.06	− **0.02**^b^	− **0.03**^b^
Range PE	− 1.64 to 1.39	− 2.32 to 1.37	− 2.19 to 1.21	− 2.54 to 1.28	− 2.29 to 1.21
MAPE ± SD	0.41 ± 0.31	0.37 ± 0.32	0.34 ± 0.3	0.36 ± 0.31	0.34 ± 0.3
MedAPE	0.35	**0.27**^a^	**0.27**^a^	**0.26**^a^	**0.26**^a^
**All eyes** (Bootstrap sample size: 2372)					
Statistics	SRK/T	Hoffer Q	Holladay I	Haigis	MM
% Rx within ± 0.5D	59.80%	68.00%	71.20%	70.10%	**76.30%**
% Rx within ± 1.0D	83.50%	95.00%	95.20%	**96.30%**^a^	**96.00%**^a^
% Rx within ± 1.5D	93.10%	**99.80%**	99.70%^a^	**99.80%**	99.50%^a^
MPE ± SD	0.05 ± 0.76	**0.0 ± 0.51**	− 0.03 ± 0.48	0.0 ± 0.49	0.0 ± 0.47
MedPE	0.17	**0.03**^b^	− 0.04	**0**^b^	− **0.02**^b^
Range PE	− 2.43 to 2.14	− 2.32 to 1.37	− 2.19 to 1.41	− 2.54 to 1.42	− 2.29 to 1.7
MAPE ± SD	0.56 ± 0.51	0.39 ± 0.32	0.37 ± 0.31	0.38 ± 0.31	0.36 ± 0.31
MedAPE	0.41	0.31	0.3	0.31	**0.28**

*% Rx* Percentage of eyes within a given range of prediction error, *MPE* Mean prediction error, *MedPE* Median prediction error, *Range PE* Range of the prediction error, *MAPE* Mean absolute prediction error, *MedAPE* Median absolute prediction error, *SD* Standard Deviation of the error.

^a^No statistically significant difference at level 0.05.

^b^No statistically significant difference from zero at level 0.05.

Significant values are in [bold].

**Table 7:** Pairwise comparison of the prediction accuracy (i.e., the percentage of eyes within a given range of prediction error) between the MM formula and each of the other four formulae (SRK/T, Hoffer Q, Holladay I and Haigis) for the different types of eyes (long, long medium, medium, short and all eyes), using the McNemar test at a statistical significance level of 5%.

Monofocal Lenstec Softec HDO			
Pairwise comparison of the prediction accuracy *p*-values for McNemar test			
**Long eyes**			
	% Rx within ± 0.5D	% Rx within ± 1.0D	% Rx within ± 1.5D
MM vs SRK/T	< **0.001**^b^	< **0.001**^b^	< **0.001**^b^
MM vs Hoffer Q	< **0.001**^b^	0.999^a^	0.999^a^
MM vs Holladay I	< **0.001**^b^	0.999^a^	0.999^a^
MM vs Haigis	**0.041**^b^	0.999^a^	0.999^a^
**Long medium eyes**			
	% Rx within ± 0.5D	% Rx within ± 1.0D	% Rx within ± 1.5D
MM vs SRK/T	< **0.001**^b^	< **0.001**^b^	< **0.001**^b^
MM vs Hoffer Q	< **0.001**^b^	0.789^a^	0.999^a^
MM vs Holladay I	0.211^a^	0.999^a^	0.999^a^
MM vs Haigis	0.864^a^	0.752^a^	0.999^a^
**Medium eyes**			
	% Rx within ± 0.5D	% Rx within ± 1.0D	% Rx within ± 1.5D
MM vs SRK/T	< **0.001**^b^	< **0.001**^b^	0.999^a^
MM vs Hoffer Q	< **0.001**^b^	< **0.001**^b^	0.999^a^
MM vs Holladay I	< **0.001**^b^	< **0.001**^b^	0.617^a^
MM vs Haigis	< **0.001**^b^	< **0.001**^b^	0.999^a^
**Short eyes**			
	% Rx within ± 0.5D	% Rx within ± 1.0D	% Rx within ± 1.5D
MM vs SRK/T	**0.003**^b^	< **0.001**^b^	< **0.001**^b^
MM vs Hoffer Q	0.585^a^	0.770^a^	*0.041*^c^
MM vs Holladay I	**0.001**^b^	0.607^a^	*0.041*^c^
MM vs Haigis	0.831^a^	< *0.001*^c^	*0.041*^c^
**All eyes**			
	% Rx within ± 0.5D	% Rx within ± 1.0D	% Rx within ± 1.5D
MM vs SRK/T	< **0.001**^b^	< **0.001**^b^	< **0.001**^b^
MM vs Hoffer Q	< **0.001**^b^	**0.013**^b^	*0.023*^c^
MM vs Holladay I	< **0.001**^b^	**0.013**^b^	0.343^a^
MM vs Haigis	< **0.001**^b^	0.391^a^	*0.023*^c^

^a^No statistically significant difference at level 0.05.

^b^MM formula outperformed at significance level 0.05.

^c^MM formula underperformed at significance level 0.05.

Significant values are in [bold, Italics].

**Table 8:** Summary statistics of the cross-validation results for each of the five formulae (SRK/T, Hoffer Q, Holladay I, Haigis and MM) for long, long medium and short eyes.

Multifocal ZEISS AT LISA tri839 MP Summary statistics of cross-validation results for long, long medium and short eyes					
**Long eyes** (Bootstrap sample size: 69)					
Statistics	SRK/T	Hoffer Q	Holladay I	Haigis	MM
% Rx within ± 0.5D	27.50%	26.10%	52.20%	50.70%	**66.70%**
% Rx within ± 1.0D	58.00%	71.00%	88.40%	88.40%	**100.00%**
% Rx within ± 1.5D	91.30%	**100.00%**^a^	**100.00%**^a^	**100.00%**^a^	**100.00%**^a^
MPE ± SD	− 0.58 ± 0.73	0.67 ± 0.5	0.54 ± 0.42	0.36 ± 0.47	0.11 ± 0.46
MedPE	− 0.58	0.77	0.43	0.5	**0.13**
Range PE	− 2.03 to 0.57	− 0.2 to 1.46	− 0.03 to 1.28	− 0.46 to 1.08	− 0.75 to 0.91
MAPE ± SD	0.78 ± 0.5	0.7 ± 0.44	0.55 ± 0.41	0.56 ± 0.21	0.39 ± 0.26
MedAPE	0.58	0.77	0.43	0.5	**0.4**
**Long medium eyes** (Bootstrap sample size: 112)					
Statistics	SRK/T	Hoffer Q	Holladay I	Haigis	MM
% Rx within ± 0.5D	44.60%	**92.00%**^a^	**87.50%**^a^	80.40%	**87.50%**^a^
% Rx within ± 1.0D	51.80%	**100.00%**^a^	**100.00%**^a^	**99.10%**^a^	**99.10%**^a^
% Rx within ± 1.5D	59.80%	**100.00%**^a^	**100.00%**^a^	**100.00%**^a^	**100.00%**^a^
MPE ± SD	− 0.99 ± 1.32	0.09 ± 0.37	− 0.05 ± 0.36	0.01 ± 0.39	− 0.03 ± 0.42
MedPE	− 0.28	**0.14**^b^	**0.12**^b^	− **0.01**^b^	**0.2**^b^
Range PE	− 2.86 to 0.56	− 0.46 to 0.96	− 0.78 to 0.51	− 0.61 to 1.02	− 1.01 to 0.56
MAPE ± SD	1.26 ± 1.06	0.31 ± 0.22	0.31 ± 0.19	0.3 ± 0.25	0.37 ± 0.21
MedAPE	0.51	0.24	0.25	**0.22**	0.3
**Short eyes** (Bootstrap sample size: 73)					
Statistics	SRK/T	Hoffer Q	Holladay I	Haigis	MM
% Rx within ± 0.5D	69.90%	61.60%	**83.60%**^a^	57.50%	**87.70%**^a^
% Rx within ± 1.0D	**100.00%**^a^	**100.00%**^a^	**100.00%**^a^	**100.00%**^a^	**100.00%**^a^
% Rx within ± 1.5D	**100.00%**^a^	**100.00%**^a^	**100.00%**^a^	**100.00%**^a^	**100.00%**^a^
MPE ± SD	0.25 ± 0.36	− 0.05 ± 0.5	− **0.04** ± **0.44**	0.22 ± 0.51	0.08 ± 0.4
MedPE	0.24	− **0.2**^b^	− **0.12**^b^	0.2	**0**^b^
Range PE	− 0.35 to 0.87	− 0.82 to 0.65	− 0.77 to 0.52	− 0.62 to 0.95	− 0.54 to 0.92
MAPE ± SD	0.37 ± 0.24	0.44 ± 0.24	0.39 ± 0.20	0.48 ± 0.28	0.37 ± 0.19
MedAPE	**0.35**^a^	**0.47**^a^	**0.44**^a^	0.44	**0.37**^a^

*% Rx* Percentage of eyes within a given range of prediction erro, *MPE* Mean prediction error, *MedPE* Median prediction error, *Range PE* Range of the prediction error, *MAPE* Mean absolute prediction error, *MedAPE* Median absolute prediction error, *SD* Standard Deviation of the error.

^a^No statistically significant difference at level 0.05.

^b^No statistically significant difference from zero at level 0.05.

Significant values are in [bold].

**Table 9:** Summary statistics of the cross-validation results for each of the five formulae (SRK/T, Hoffer Q, Holladay I, Haigis and MM) for medium and all eyes.

Multifocal ZEISS AT LISA tri839 MP Summary statistics of cross-validation results for medium and all eyes					
**Medium eyes** Bootstrap sample size: 846					
Statistics	SRK/T	Hoffer Q	Holladay I	Haigis	MM
% Rx within ± 0.5D	76.70%	76.50%	**81.20%**^a^	74.50%	**80.60%**^a^
% Rx within ± 1.0D	96.60%^a^	97.60%	97.60%	**98.60%**	96.60%^a^
% Rx within ± 1.5D	**100.00%**^a^	**100.00%**^a^	**100.00%**^a^	**100.00%**^a^	**100.00%**^a^
MPE ± SD	0.15 ± 0.4	− 0.01 ± 0.42	− 0.08 ± 0.39	− 0.03 ± 0.41	0.01 ± 0.42
MedPE	0.11	− **0.01**^b^	− 0.1	− 0.03	− **0.03** ^b^
Range PE	− 0.78 to 1.39	− 0.84 to 1.36	− 0.94 to 1.22	− 0.87 to 1.31	− 0.95 to 1.33
MAPE ± SD	0.32 ± 0.27	0.33 ± 0.26	0.31 ± 0.24	0.32 ± 0.27	0.33 ± 0.26
MedAPE	**0.24**^a^	**0.27**^a^	0.24	**0.23**^a^	**0.29**^a^
**All eyes** Bootstrap sample size: 1100					
Statistics	SRK/T	Hoffer Q	Holladay I	Haigis	MM
% Rx within ± 0.5D	69.90%	73.90%	**80.20%**^a^	72.50%	**80.90%**^a^
% Rx within ± 1.0D	89.80%	**96.40%**^a^	**97.50%**^a^	**98.10%**^a^	**97.30%**^a^
% Rx within ± 1.5D	95.40%	**100.00%**^a^	**100.00%**^a^	**100.00%**^a^	**100.00%**^a^
MPE ± SD	− 0.01 ± 0.69	0.04 ± 0.45	− 0.04 ± 0.42	0.01 ± 0.44	0.01 ± 0.42
MedPE	0.07	**0.03**^b^	− 0.03	**0**^b^	**0**^b^
Range PE	− 2.86 to 1.39	− 0.84 to 1.46	− 0.94 to 1.28	− 0.87 to 1.31	− 1.01 to 1.33
MAPE ± SD	0.45 ± 0.53	0.36 ± 0.29	0.33 ± 0.26	0.34 ± 0.27	0.34 ± 0.25
MedAPE	0.28	0.28 ^a^	0.26	**0.25**^a^	0.31^a^

*% Rx* Percentage of eyes within a given range of prediction error, *MPE* Mean prediction error, *MedPE* Median prediction error, *Range PE* Range of the prediction error, *MAPE* Mean absolute prediction error, *MedAPE* Median absolute prediction error, *SD* Standard Deviation of the error.

^a^No statistically significant difference at level 0.05.

^b^No statistically significant difference from zero at level 0.05.

Significant values are in [bold].

**Table 10:** Pairwise comparison of the prediction accuracy (i.e., the percentage of eyes within a given range of prediction error) between the MM formula and each of the other four formulae (SRK/T, Hoffer Q, Holladay I and Haigis) for the different types of eyes (long, long medium, medium, short and all eyes), using the McNemar test at a statistical significance level of 5%.

Multifocal ZEISS AT LISA tri839 MP			
**Pairwise comparison of the prediction accuracy** *p*-values for McNemar test			
**Long eyes**			
	% Rx within ± 0.5D	% Rx within ± 1.0D	% Rx within ± 1.5D
MM vs SRK/T	< **0.001**^b^	< **0.001**^b^	**0.041**^b^
MM vs Hoffer Q	< **0.001**^b^	< **0.001**^b^	0.999^a^
MM vs Holladay I	< **0.001**^b^	**0.013**^b^	0.999^a^
MM vs Haigis	< **0.001**^b^	**0.013**^b^	0.999^a^
**Long medium eyes**			
	% Rx within ± 0.5D	% Rx within ± 1.0D	% Rx within ± 1.5D
MM vs SRK/T	< **0.001**^b^	< **0.001**^b^	< **0.001**^b^
MM vs Hoffer Q	0.383^a^	0.999^a^	0.999^a^
MM vs Holladay I	0.999^a^	0.999^a^	0.999^a^
MM vs Haigis	**0.013**^b^	0.999^a^	0.999^a^
**Medium eyes**			
	% Rx within ± 0.5D	% Rx within ± 1.0D	% Rx within ± 1.5D
MM vs SRK/T	**0.001**^b^	0.999 ^a^	0.999^a^
MM vs Hoffer Q	**0.006**^b^	*0.008* ^c^	0.999^a^
MM vs Holladay I	0.699^a^	*0.008* ^c^	0.999^a^
MM vs Haigis	< **0.001**^b^	< *0.001* ^c^	0.999^a^
**Short eyes**			
	% Rx within ± 0.5D	% Rx within ± 1.0D	% Rx within ± 1.5D
MM vs SRK/T	**0.002**^b^	0.999^a^	0.999^a^
MM vs Hoffer Q	**0.002**^b^	0.999^a^	0.999^a^
MM vs Holladay I	0.646^a^	0.999^a^	0.999^a^
MM vs Haigis	< **0.001**^b^	0.999^a^	0.999^a^
**All eyes**			
	% Rx within ± 0.5D	% Rx within ± 1.0D	% Rx within ± 1.5D
MM vs SRK/T	< **0.001**^b^	< **0.001**^b^	< **0.001**^b^
MM vs Hoffer Q	< **0.001**^b^	0.100^a^	0.999^a^
MM vs Holladay I	0.580^a^	0.814^a^	0.999^a^
MM vs Haigis	< **0.001**^b^	0.124^a^	0.999^a^

^a^No statistically significant difference at level 0.05.

^b^MM formula outperformed at significance level 0.05.

^c^MM formula underperformed at significance level 0.05.

Significant values are in [bold, Italics].

For the IOL model Monofocal Alcon AcrySofIQ SN60WF, the results in Tables 2, 3, 4, show that, overall, the MM formula outperformed the other four formulae. For long eyes, the MM formula and the Haigis formula outperformed the other formulae in terms of prediction accuracy, i.e., the percentage of eyes with the prediction error within the range ± 0.5D, ± 1.0D, and ± 1.5D, respectively. On the other hand, the MM formula had the lowest median absolute prediction error. For long medium eyes, the MM formula was the second best behind the Holladay I formula in terms of prediction accuracy. However, the MM formula has the lowest median absolute prediction error. For short eyes, overall, the MM formula was the second best in terms of prediction accuracy, but with the lowest median absolute prediction error. For medium and all eyes, overall, the MM formula outperformed the other formulae, with the highest prediction accuracy and the lowest median absolute prediction error.

For the IOL model Monofocal Lenstec Softec HDO, the results in Tables 5, 6, 7 show that, overall, the MM formula performed better compared to the other formulae. For long and long medium eyes, MM formula achieved the highest performance in terms of prediction accuracy and low median absolute prediction error. For short eyes, the MM formula was outperformed by the Haigis formula in terms of prediction accuracy whereas the Hoffer Q formula has the lowest median absolute prediction error. For medium and all eyes, overall, the MM formula outperformed the other formulae with the highest prediction accuracy and the lowest median absolute prediction error.

For the IOL model Multifocal ZEISS AT LISA tri839 MP, the results in Tables 8, 9, 10, show that, overall, the MM formula outperformed the other formulae. For long, long medium and short eyes, the MM formula achieved the highest performance in terms of prediction accuracy, and it has the lowest median absolute prediction error for long and short eyes. For medium and all eyes, overall, the MM performed similarly to the best performing formula.

## Discussion

Overall, the results reported in this study, which are comparable to those presented in the literature^9–11,13,14,26^, show that the MM formula outperformed  the four commonly used modern formulae in terms of median absolute error as well as prediction accuracy, in particular within the range ± 0.5 D and ± 1 D, for various ranges of axial length. This robustness of the MM formulae enables the method to cope with the variation of the axial length (L), the pre-operative ACD and keratometry readings (K_1_ and K_2_), hence mitigating the impact of measurement errors for these variables. Using all lens models, for both average eyes as well as more challenging eyes (i.e., short, long medium and long eyes), the results of the post-refractive outcomes for the MM formula are overall superior compared to the other four formulae.  However, all the formulae exceeded the benchmark of 85% of refraction within the range ± 1D of the prediction, recommended by Gale et al.^27^, except some cases for the SRK/T formulae. The discrepancy between our results and other findings in the literature, for instance the good performance of the SRK/T formula for eyes with long axial length may be attributed to the cross-validation approach, which considered many training and test sets, and this variation may be reflected in the optimized A_constant_.

The most well-known formula using machine learning techniques, is probably the Hill-RBF method^8^, which is purely data driven, and used artificial neural network to estimate directly the IOL power. The assessment of the formula is based on the hold-out method, (i.e., using one training and one test set). However, the performance of machine learning-based predictive models may depend on the samples used to train and test the model, and the cross-validation is the most appropriate approach to assess the generalization of the model. In contrast with this method, the approach presented in this paper used an ensemble of regression models to provide a more accurate prediction of the ELP, thus it combines both geometric optics and machine learning. The BART^9^ is another learning-based formula, which combines the Wang-Koch modified SRK/T formula^28^ and Bayesian additive regression trees to estimate the IOL power. The assessment of the formula using five random out-of-sample validations yields a “median absolute refraction error” and “standard deviation of the refractive error” of 0.137D and 0.204D, respectively. Furthermore, the dataset used in the analysis consists of a combination of various lens models and the aggregated results for all eyes were provided. Another learning-based formula for IOL power calculation is the Karmona formula^10^. The assessment of the formula on a single test set of 52 eyes, and using a mix of ten models of monofocal lenses, yields a mean absolute error of 0.24D, a median absolute error of 0.18, percentages of refraction within the ranges ± 0.5D and ± 1D of 90.38% and 100%, respectively.

However, it is well known that the performance of IOL power calculation formulae may vary depending on the lens model as well as the eye characteristics, in particular the axial length of the eye. The use of cross-validation as well as the stratification of the data by axial length of the eye would provide more insight on the performance of these formulae.

Since learning-based IOL calculation formulae are essentially data driven predictive models, the most appropriate approach to compare their performance is to assess them using the same dataset. On the other hand, their implementation details are not available; hence, a direct comparison of performance metrics from various studies, using different datasets, may be misleading. Nevertheless, the assessment of the MM formula using the cross-validation concept and across eyes stratified by their axial length as well as various lens models (monofocal and multifocal) highlighted its robustness. Furthermore, the MM formula is quite flexible and can accommodate as much predictor variables available and then analyze them to identify the most relevant ones for each surgeon/IOL pair. However, the MM formula has some limitations, which are inherent to machine learning techniques. Although machine learning techniques have demonstrated many successful applications in various fields, they have some fundamental limitations, which could hinder their effectiveness in some real-word scenarios. For instance, the MM formula requires a large amount of structured training dataset in order to learn patterns effectively. Furthermore, the MM formula encode correlation and not causation, and the accuracy of its prediction revolve around the quality of the data.

## Supplementary Information

Supplementary Information.

## References

[1] Haigis W, Lege B, Miller N, Schneider B (2000). Comparison of immersion ultrasound biometry and partial coherence interferometry for intraocular lens calculation according to Haigis. Graefes Arch. Clin. Exp. Ophthalmol..

[2] Hoffer KJ (1993). The Hoffer Q formula: a comparison of theoretic and regression formulas. J. Cataract. Refract. Surg..

[3] Holladay JT (1988). A three-part system for refining intraocular lens power calculations. J. Cataract. Refract. Surg..

[4] Retzlaff JA, Sanders DR, Kraff MC (1990). Development of the SRK/T intraocular lens implant power calculation formula. J. Cataract. Refract. Surg..

[5] Olsen T (2006). Prediction of the effective postoperative (intraocular lens) anterior chamber depth. J. Cataract. Refract. Surg..

[6] Barrett, G. D. Barrett Universal II Formula. Available at https://calc.apacrs.org/barrett_universal2105/ Last accessed 30th July 2021.

[7] Clarke GP, Burmeister J (1997). Comparison of intraocular lens computations using a neural network versus the Holladay formula. J. Cataract. Refract. Surg..

[8] Hill, W. E. IOL Power Selection by Pattern Recognition; *ASCRS EyeWorld Corporate Education*; ASCRS (2016).

[9] González DC, Bautista CP (2021). Accuracy of a new intraocular lens power calculation method based on artificial intelligence. Eye.

[10] Clarke GP, Kapelner A (2020). The bayesian additive regression trees formula for safe machine learning-based intraocular lens predictions. Front Big Data..

[11] Cooke DL, Cooke TL (2016). Comparison of 9 intraocular lens power calculation formulas. J. Cataract. Refract. Surg..

[12] Hoffer KJ, Savini G (2017). IOL power calculation in short and long eyes. Asia-Pacific J. Ophthalmol..

[13] Nihalani BR, VanderVeen DK (2010). Comparison of intraocular lens power calculation formulae in pediatric eyes. Ophthalmology.

[14] Shammas HJ, Shammas MC (2007). No-history method of intraocular lens power calculation for cataract surgery after myopic laser in situ keratomileusis. J. Cataract. Refract. Surg..

[15] Wang K, Hu CY, Chang SW (2008). Intraocular lens power calculation using the IOL Master and various formulas in eyes with long axial length. J. Cataract. Refract. Surg..

[16] Vasavada V (2016). Comparison of IOL power calculation formulae for pediatric eyes. Eye.

[17] Sanders DR, Retzlaff J, Kraff MC (1988). Comparison of the SRK II formula and other second-generation formulae. J. Cataract. Refract. Surg..

[18] Thompson JT, Maumenee AE, Baker CC (1984). A new posterior chamber intraocular lens formula for axial myopes. Ophthalmology.

[19] Donzis PB, Kastl PR, Gordon RA (1985). An intraocular lens formula for short, normal and long eyes. CLAO J..

[20] Thijssen JM (1975). The emmetropic and the iseikonic implant lens: Computer calculation of the refractive power and its accuracy. Ophthalmologica.

[21] Colenbrander MC (1973). Calculation of the power of an iris clip lens for distant vision. Br. J. Ophthalmol..

[22] van der Heijde GL (1976). The optical correction of unilateral aphakia. Trans. Sect. Ophthalmo. Am. Acad. Ophthalmol. Otolaryngol..

[23] Binkhorst RD (1975). The optical design of intraocular lens implants. Ophthalmic Surg..

[24] Fyodorov SN, Galin MA, Linksz A (1975). Calculation of the optical power of intraocular lenses. Invest Ophthalmol..

[25] ULIB Database - http://ocusoft.de/ulib/; Last accessed 30^th^ July 2021.

[26] Shammas, J. H. (ed.) Intraocular lens power calculations. SLACK Incorporated, 6900 Grove Road, Thorofare, NJ 08086 (2003).

[27] Gale RP, Saldana M, Johnston RL, Zuberbuhler B, McKibbin M (2009). Benchmark standards for refractive outcomes after NHS cataract surgery. Eye.

[28] Wang L, Shirayama M, Ma XJ, Kohnen T, Koch DD (2011). Optimizing intraocular lens power calculations in eyes with axial lengths above 25.0 mm. J. Cataract. Refract. Surg..

