# Supplementary material for: An ensemble-based approach for estimating personalized intraocular lens power
Source: Sci Rep. 2021 Nov 25;11:22961. doi: 10.1038/s41598-021-02288-x (PMC8617163; doi:10.1038/s41598-021-02288-x)
Supplement: Supplementary file 1 — Supplementary Information. [file 41598_2021_2288_MOESM1_ESM.docx]

**Table S1:** Distribution of eyes implanted with the three lens models by axial length (AL).

| **Distribution of eyes by axial length (AL)** | | | | | | | |
| --- | --- | --- | --- | --- | --- | --- | --- |
| **Lens model** | **Sample Size**  (Eyes)  One eye per  Patient | **Long eyes**  AL>26mm | **Long medium eyes**  24.5mm < AL  and  AL<=26mm | **Medium eyes**  22mm<= AL  and  AL<=24.5mm | **Short eyes**  AL<22mm | **All eyes** |  |
| **Monofocal Alcon AcrySofIQ SN60WF** | 265 | 11% | 18% | 62% | 9% | 100% |  |
| **Monofocal**  **Lenstec Softec HDO** | 256 | 5% | 13% | 72% | 10% | 100% |  |
| **Multifocal**  **ZEISS AT LISA tri839 MP** | 160 | 6% | 10% | 77% | 7% | 100% |  |

**Table S2:** Summary statistics of the optimized IOL constants estimated from the training sets and used for the test sets during the cross-validation process. Note that at each iteration of the cross-validation, new optimized IOL constants are generated. SD=Standard Deviation.

|  | **Summary statistics - Optimized IOL constants** | | | | |
| --- | --- | --- | --- | --- | --- |
| **IOL model** | **IOL constants** | **Mean**±**SD** | **Median** | **Range** |  |
| **Monofocal**  **Alcon AcrySofIQ SN60WF**  (Bootstrap sample size: 2486) | SRK/T (A-Constant) | 118.861±0.021 | 118.87 | 118.808 to 118.919 |  |
|  | Hoffer Q (pACD) | 5.738±0.007 | 5.736 | 5.722 to 5.764 |  |
|  | Holladay I (SF) | 2.065±0.01 | 2.066 | 2.037 to 2.098 |  |
|  | Haigis (a_0_) | -0.966±0.147 | -0.952 | -1.322 to -0.559 |  |
|  | Haigis (a_1_) | 0.293±0.023 | 0.293 | 0.24 to 0.379 |  |
|  | Haigis (a_2_) | 0.222±0.008 | 0.222 | 0.2 to 0.238 |  |
| **Monofocal Lenstec Softec HDO**  (Bootstrap sample size: 2372) | SRK/T (A-Constant) | 117.976±0.018 | 117.979 | 117.902 to 118.018 |  |
|  | Hoffer Q (pACD) | 5.35±0.008 | 5.35 | 5.328 to 5.377 |  |
|  | Holladay I (SF) | 1.614±0.008 | 1.615 | 1.595 to 1.633 |  |
|  | Haigis (a_0_) | -1.207±0.168 | -1.186 | -1.811 to -0.758 |  |
|  | Haigis (a_1_) | 0.252±0.028 | 0.254 | 0.192 to 0.315 |  |
|  | Haigis (a_2_) | 0.22±0.009 | 0.22 | 0.194 to 0.249 |  |
| **Multifocal**  **ZEISS AT LISA tri839 MP**  (Bootstrap sample size: 1100) | SRK/T (A-Constant) | 118.73±0.02 | 118.727 | 118.665 to 118.785 |  |
|  | Hoffer Q (pACD) | 5.639±0.008 | 5.64 | 5.618 to 5.659 |  |
|  | Holladay I (SF) | 1.97±0.013 | 1.971 | 1.934 to 1.992 |  |
|  | Haigis (a_0_) | -0.146±0.299 | -0.171 | -0.968 to 0.687 |  |
|  | Haigis (a_1_) | 0.272±0.05 | 0.28 | 0.13 to 0.442 |  |
|  | Haigis (a_2_) | 0.184±0.016 | 0.185 | 0.129 to 0.224 |  |

**Table S3a:** Comparison of the median prediction error to zero for each of the five formulae (SRK/T, Hoffer Q, Holladay I, Haigis and MM) and for the different types of eyes (long, long medium, medium, short and all eyes), using Wilcoxon (1 sample) signed rank test at a statistical significance level of 5%.

| **Monofocal Alcon AcrySofIQ SN60WF** | | | | | |
| --- | --- | --- | --- | --- | --- |
| **Comparison of the median prediction error to zero**  p-values for Wilcoxon (1 sample) signed rank test | | | | | |
|  | SRK/T | Hoffer Q | Holladay I | Haigis | MM |
| Long eyes | < 0.001 | < 0.001 | < 0.001 | < 0.001 | 0.4302^a^ |
| Long medium eyes | < 0.001 | < 0.001 | 0.001 | 0.7507^a^ | < 0.001 |
| Medium eyes | < 0.001 | 0.0007 | < 0.001 | 0.0011 | 0.0915^a^ |
| Short eyes | 0.0043 | < 0.001 | < 0.001 | 0.4602^a^ | < 0.001 |
| All eyes | < 0.001 | < 0.001 | < 0.001 | 0.8405^a^ | 0.5777^a^ |
| ^a^ No statistically significant difference from zero at level 0.05 | | | | | |

**Table S3b:** Top - Comparison of the median absolute prediction errors of the five formulae (SRK/T, Hoffer Q, Holladay I, Haigis and MM) for the different types of eyes (long, long medium, medium, short and all eyes), using the Friedman test at a statistical significance level of 5%.

Bottom – Pairwise comparison of the median absolute prediction errors between the MM formula and each of the other four formulae (SRK/T, Hoffer Q, Holladay I and Haigis) for the different types of eyes (long, long medium, medium, short and all eyes), using the Wilcoxon test at a statistical significance level of 5%.

| **Monofocal Alcon AcrySofIQ SN60WF** | | | | | |
| --- | --- | --- | --- | --- | --- |
| **Comparison of the median absolute prediction error for the five formulae**  p-values for Friedman test | | | | | |
|  | Long eyes | Long medium  eye | Medium eyes | Short eyes | All eyes |
| p-Values | < 0.001 | < 0.001 | < 0.001 | < 0.001 | < 0.001 |
| **Pairwise comparison of the median absolute prediction error**  p-values for Wilcoxon (2 paired samples) signed rank test | | | | | |
| Formulae pair | Long eyes | Long medium  Eye | Medium eyes | Short eyes | All eyes |
| MM vs SRK/T | < 0.001^b^ | < 0.001^b^ | < 0.001^b^ | 0.901^a^ | < 0.001^b^ |
| MM vs Hoffer Q | < 0.001^b^ | < 0.001^b^ | < 0.001^b^ | < 0.001^b^ | < 0.001^b^ |
| MM vs Holladay I | < 0.001^b^ | 0.007^b^ | < 0.001^b^ | < 0.001^b^ | < 0.001^b^ |
| MM vs Haigis | < 0.001^b^ | 0.001^b^ | < 0.001^b^ | 0.001^b^ | < 0.001^b^ |
| ^a^ No statistically significant difference at level 0.05  ^b^ MM formula underperformed at significance level 0.05 | | | | | |

**Table S3c:** Comparison of the prediction accuracy (i.e., the percentage of eyes within a given range of prediction error) of the five formulae (SRK/T, Hoffer Q, Holladay I, Haigis and MM) for the different types of eyes (long, long medium, medium, short and all eyes), using the Cochran Q test, at a statistical significance level of 5%.

| **Monofocal Alcon AcrySofIQ SN60WF** | | | |
| --- | --- | --- | --- |
| **Comparison of the prediction accuracy for the five formulae**  p-values for Cochran’s Q test | | | |
|  | % Rx within ±0.5D | % Rx within ±1.0D | % Rx within ±1.5D |
| Long eyes | < 0.001 | < 0.001 | < 0.001 |
| Long medium eyes | < 0.001 | < 0.001 | < 0.001 |
| Medium eyes | < 0.001 | < 0.001 | 0.063^a^ |
| Short eyes | < 0.001 | < 0.001 | 0.732^a^ |
| All eyes | < 0.001 | < 0.001 | < 0.001 |
| ^a^ No statistically significant difference at level 0.05 | | | |

**Table S4a:** Comparison of the median prediction error to zero for each of the five formulae (SRK/T, Hoffer Q, Holladay I, Haigis and MM) and for the different types of eyes (long, long medium, medium, short and all eyes), using Wilcoxon (1 sample) signed rank test at a statistical significance level of 5%.

| **Monofocal Lenstec Softec HDO** | | | | | |
| --- | --- | --- | --- | --- | --- |
| **Comparison of the median prediction error to zero**  p-values for Wilcoxon (1 sample) signed rank test | | | | | |
|  | SRK/T | Hoffer Q | Holladay I | Haigis | MM |
| Long eyes | < 0.001 | < 0.001 | < 0.001 | 0.039 | 0.092^a^ |
| Long medium eyes | < 0.001 | < 0.001 | 0.169^a^ | 0.037 | 0.064^a^ |
| Medium eyes | < 0.001 | 0.367^a^ | < 0.001 | 0.253^a^ | 0.164^a^ |
| Short eyes | 0.0043 | < 0.001 | 0.410^a^ | 0.091^a^ | 0.066^a^ |
| All eyes | < 0.001 | 0.127^a^ | < 0.001 | 0.275^a^ | 0.701^a^ |
| ^a^ No statistically significant difference from zero at level 0.05 | | | | | |

**Table S4b:** Top - Comparison of the median absolute prediction errors of the five formulae (SRK/T, Hoffer Q, Holladay I, Haigis and MM) for the different types of eyes (long, long medium, medium, short and all eyes), using the Friedman test at a statistical significance level of 5%.

Bottom – Pairwise comparison of the median absolute prediction errors between the MM formula and each of the other four formulae (SRK/T, Hoffer Q, Holladay I and Haigis) for the different types of eyes (long, long medium, medium, short and all eyes), using the Wilcoxon test at a statistical significance level of 5%.

| **Monofocal Lenstec Softec HDO** | | | | | |
| --- | --- | --- | --- | --- | --- |
| **Comparison of the median absolute prediction error for the five formulae**  p-values for Friedman test | | | | | |
|  | Long eyes | Long medium  eye | Medium eyes | Short eyes | All eyes |
| p-Values | < 0.001 | < 0.001 | < 0.001 | < 0.001 | < 0.001 |
| **Pairwise comparison of the median absolute prediction error**  p-values for Wilcoxon (2 paired samples) signed rank test | | | | | |
| Formulae pair | Long eyes | Long medium  Eye | Medium eyes | Short eyes | All eyes |
| MM vs SRK/T | < 0.001^b^ | < 0.001^b^ | < 0.001^b^ | < 0.001^b^ | < 0.001^b^ |
| MM vs Hoffer Q | < 0.001^b^ | < 0.001^b^ | 0.785^a^ | 0.032^c^ | < 0.001^b^ |
| MM vs Holladay I | < 0.001^b^ | < 0.001^b^ | 0.785^a^ | < 0.001^b^ | < 0.001^b^ |
| MM vs Haigis | < 0.001^b^ | 0.002^b^ | 0.999^a^ | 0.047^c^ | < 0.001^b^ |
| ^a^ No statistically significant difference at level 0.05  ^b^ MM formula underperformed at significance level 0.05  ^c^ MM formula underperformed at significance level 0.05 | | | | | |

**Table S4c:** Comparison of the prediction accuracy (i.e., the percentage of eyes within a given range of prediction error) of the five formulae (SRK/T, Hoffer Q, Holladay I, Haigis and MM) for the different types of eyes (long, long medium, medium, short and all eyes), using the Cochran Q test, at a statistical significance level of 5%.

| **Monofocal Lenstec Softec HDO** | | | |
| --- | --- | --- | --- |
| **Comparison of the prediction accuracy for the five formulae**  p-values for Cochran’s Q test | | | |
|  | % Rx within ±0.5D | % Rx within ±1.0D | % Rx within ±1.5D |
| Long eyes | < 0.001 | < 0.001 | < 0.001 |
| Long medium eyes | < 0.001 | < 0.001 | < 0.001 |
| Medium eyes | < 0.001 | < 0.001 | 0.111^a^ |
| Short eyes | < 0.001 | < 0.001 | < 0.001 |
| All eyes | < 0.001 | < 0.001 | < 0.001 |
| ^a^ No statistically significant difference at level 0.05 | | | |

**Table S5a:** Comparison of the median prediction error to zero for each of the five formulae (SRK/T, Hoffer Q, Holladay I, Haigis and MM) and for the different types of eyes (long, long medium, medium, short and all eyes), using Wilcoxon (1 sample) signed rank test at a statistical significance level of 5%.

| **Multifocal ZEISS AT LISA tri839 MP** | | | | | |
| --- | --- | --- | --- | --- | --- |
| **Comparison of the median prediction error to zero**  p-values for Wilcoxon (1 sample) signed rank test | | | | | |
|  | SRK/T | Hoffer Q | Holladay I | Haigis | MM |
| Long eyes | < 0.001 | < 0.001 | < 0.001 | < 0.001 | 0.027 |
| Long medium eyes | < 0.001 | 0.122^a^ | 0.102^a^ | 0.556^a^ | 0.736^a^ |
| Medium eyes | < 0.001 | 0.316^a^ | < 0.001 | 0.017 | 0.557^a^ |
| Short eyes | < 0.001 | 0.543^a^ | 0.662^a^ | 0.002 | 0.149^a^ |
| All eyes | < 0.001 | 0.060^a^ | < 0.001 | 0.803^a^ | 0.592^a^ |
| ^a^ No statistically significant difference from zero at level 0.05 | | | | | |

**Table S5b:** Top - Comparison of the median absolute prediction errors of the five formulae (SRK/T, Hoffer Q, Holladay I, Haigis and MM) for the different types of eyes (long, long medium, medium, short and all eyes), using the Friedman test at a statistical significance level of 5%.

Bottom – Pairwise comparison of the median absolute prediction errors between the MM formula and each of the other four formulae (SRK/T, Hoffer Q, Holladay I and Haigis) for the different types of eyes (long, long medium, medium, short and all eyes), using the Wilcoxon test at a statistical significance level of 5%.

| **Multifocal ZEISS AT LISA tri839 MP** | | | | | |
| --- | --- | --- | --- | --- | --- |
| **Comparison of the median absolute prediction error for the five formulae**  p-values for Friedman test | | | | | |
|  | Long eyes | Long medium  eye | Medium eyes | Short eyes | All eyes |
| p-Values | 0.005 | < 0.001 | < 0.001 | < 0.001 | < 0.001 |
| **Pairwise comparison of the median absolute prediction error**  p-values for Wilcoxon (2 paired samples) signed rank test | | | | | |
| Formulae pair | Long eyes | Long medium  Eye | Medium eyes | Short eyes | All eyes |
| MM vs SRK/T | < 0.001^b^ | < 0.001^b^ | 0.290^a^ | 0.811^a^ | < 0.001 ^c^ |
| MM vs Hoffer Q | < 0.001^b^ | 0.002 ^c^ | 0.340^a^ | 0.060^a^ | 0.327^a^ |
| MM vs Holladay I | 0.023^b^ | < 0.001 ^c^ | 0.001^b^ | 0.159^a^ | 0.044 ^c^ |
| MM vs Haigis | < 0.001^b^ | < 0.001 ^c^ | 0.132^a^ | 0.012^b^ | 0.766^a^ |
| ^a^ No statistically significant difference at level 0.05  ^b^ MM formula outperformed at significance level 0.05  ^c^ MM formula underperformed at significance level 0.05 | | | | | |

**Table S5c:** Comparison of the prediction accuracy (i.e., the percentage of eyes within a given range of prediction error) of the five formulae (SRK/T, Hoffer Q, Holladay I, Haigis and MM) for the different types of eyes (long, long medium, medium, short and all eyes), using the Cochran Q test, at a statistical significance level of 5%.

| **Multifocal ZEISS AT LISA tri839 MP** | | | |
| --- | --- | --- | --- |
| **Comparison of the prediction accuracy for the five formulae**  p-values for Cochran’s Q test | | | |
|  | % Rx within ±0.5D | % Rx within ±1.0D | % Rx within ±1.5D |
| Long eyes | < 0.001 | < 0.001 | 0.002 |
| Long medium eyes | 0.002 | < 0.001 | 0.002 |
| Medium eyes | < 0.001 | < 0.001 | 0.999^a^ |
| Short eyes | < 0.001 | 0.999^a^ | 0.999^a^ |
| All eyes | < 0.001 | < 0.001 | 0.002 |
| ^a^ No statistically significant difference at level 0.05 | | | |
